# miR-21 Is a Promising Novel Biomarker for Lymph Node Metastasis in Patients with Gastric Cancer

**DOI:** 10.1155/2012/640168

**Published:** 2012-06-27

**Authors:** Yuejuan Xu, Jue Sun, Jianhua Xu, Qi Li, Yuewu Guo, Qiang Zhang

**Affiliations:** ^1^Department of Oncology, The Second Hospital of Nanjing, South East University, Nanjing 210003, China; ^2^Department of Oncology, Putuo Hospital, Shanghai University of Traditional Chinese Medicine, Shanghai 200062, China; ^3^Department of Oncology, Shanghai Sixth People's Hospital, Shanghai Jiao Tong University, Shanghai 200233, China

## Abstract

*Background*. Gastric cancer (GC) is an important malignant disease around the world. Abnormalities of microRNAs (miRNAs) have been implicated in carcinogenesis of various cancers. In the present study, we examined miR-21 expression in human gastric cancer with lymph node metastasis and attempted to uncover its relationship with clinicopathologic data, especially with lymph node metastasis. *Materials and Methods*. The expression levels of miR-21 in the tumor specimens of GC patients were quantified by RT-PCR. The correlation between miR-21 level and multiple clinicopathological factors was then examined by Mann-Whitney test, Kaplan-Meier survival analysis, and operating characteristic (ROC) analysis. *Results*. The expression level of miR-21 was higher in GC patients with lymph node metastasis than in those without lymph node metastasis (*P* < 0.05). Expression level of miR-21 was significantly correlated with histologic type, T stage, lymph node metastasis and pTNM stage. The overall survival rates in GC patients with low upregulated miR-21 expression were significantly higher than those with high upregulated miR-21 (*P* < 0.05). *Conclusion*. A close association is implicated between the elevated miR-21and lymph node metastasis, which could potentially be exploited as a practical biomarker for lymph node metastasis in patients with GC.

## 1. Introduction

Gastric cancer (GC) is the fourth most prevalent malignant cancer worldwide and is the second most frequent cause of cancer death [1]. Despite many advances made in GC therapy, the prognosis for patients with GC remains unsatisfying. Because of early detection in screening programmes in Japan, survival is good (52%), whereas survival in the USA, Europe, and China generally is only 20–25% for the delayed diagnosis [2]. Five-year survival rate for advanced or metastatic gastric cancer is nearly 5–20%, with median overall survival being less than 1 year [3, 4]. Therefore, it is necessary to find prognostic markers that could accurately indicate biological characteristics of GC and supply the evidence for early diagnosis and predicting the clinical outcome so as to improve the clinical management of GC patients. With the advances in diagnostic and operative technologies, surgical decisions have been made based on tumor stage [5, 6]. In order to tailor surgical therapy, it is necessary to assess clinical stage and depth of tumor invasion. However, the method for prediction of lymph node metastasis, another important determinant for prognosis, has not yet been well established. 

MicroRNAs (miRNAs), a species of small noncoding RNA of about 21–23 nucleotides, could interact with their target mRNAs to interfere with the translation by promoting mRNA degradation or to block translation by binding to partial homology to target mRNA in the 39-untranslated region [7, 8]. It has been reported that miRNAs play important roles in various human biological processes such as metabolism, differentiation, cell proliferation, and apoptosis. Abnormalities of miRNA are suggested in carcinogenesis of various cancers, indicating that miRNAs could be used as molecular biomarkers for diagnosis of cancer and prediction of prognosis [9–12]. MiR-21 has been reported to be elevated in multiple human solid tumors including lung, breast, stomach, prostate, colon, and pancreatic cancer and their respective normal adjacent tissue. Meanwhile, serum miR-21 was also found to be overexpressed in many cancers such as diffuse large B-cell lymphoma, ovarian cancer, prostate cancer, or breast cancer. Evidence supports the hypothesis that miR-21 is a central oncomiR [13–16]. 

In the present study, we explore miR-21 expression and its correlation with clinicopathological factors in gastric cancer. Furthermore, we also determine whether miR-21 expression in lymph node might be a molecular biomarker for predicting the lymph node metastasis of GC patients.

## 2. Materials and Methods

### 2.1. Patients and Samples

Tumor specimens and normal control tissues were collected from 86 patients who underwent surgical treatment for histologically proven gastric adenocarcinoma at the Department of Surgery, Nanjing Second People's Hospital, Shanghai Sixth People's Hospital, and Shanghai Putuo Hospital, from January 2006 to December 2008. None of the patients had been administered by chemotherapy or radiotherapy prior to undergoing surgical resection. Metastatic lymph nodes were also harvested during gastrectomy. Clinical stage of GC was assessed on the basis of the tumor node metastasis (TNM) classification system recommended by the International Union against Cancer. For accurate N staging, more than 15 lymph nodes in one patient were collected by means of a careful manual palpation. Demographic and clinicopathological details of patients were collected from electronic patient records. The study was approved by the Institutional Review Board of Nanjing Second People's Hospital, Shanghai Sixth People's Hospital, and Shanghai Putuo Hospital. *Written informed* consent was *obtained* from each participant.

### 2.2. RNA Extraction and qRT-PCR

Total RNA was extracted from frozen specimens using Trizol (Invitrogen) following the manufacturer's guide. Total RNA was eluted in 100 mL and stored at −20°C. 5 mL of RNA was used to measure the expression of miR-21 by quantitative RT-PCR (qRT-PCR) with the TaqManH miRNA reverse transcription kit and the TaqManH miRNA assay-specific RT primers for miR-21 according to the instructions of the manufacturer (Applied Biosystems, Foster City, CA). The expression of Let-7a was used as internal control. Real-time PCR was performed with 3 mL of each cDNA on a StepOnePlus Real-Time PCR System (Applied Biosystems, Foster City, CA) in duplicates. *C*_*T*_ value is defined as the number of PCR cycles at which the fluorescent signal crosses the threshold. The difference of *C*_*T*_ values between internal control and miR-21was presented as −Δ*C*_*T*_. ΔΔ*C*_*T*_ is the difference of Δ*C*_*T*_ values between paired specimens. 2^ΔΔ*CT*^ represents the exponential value of Δ*C*_*T*_, which means fold change in expression.

### 2.3. Statistical Analysis

The correlation between up-regulated miR-21 and different clinical factors was assessed by Mann-Whitney analysis, paired *t*-test linear regression, and Kaplan-Meier survival analysis. To determine to which extent the obtained −Δ*C*_*T*_ value of miR-21 could efficiently separate different clinical subsettings, operating characteristic (ROC) analysis was generated, and the sensitivity and specificity of the optimum cut-off point were defined as those values that maximized the area under the ROC curve (AUC). Data analysis was done using R software statistical environment (version 2.14.1; R Development Core Team, Vienna, Austria). All statistical tests were two-sided, and *P* value of less than .05 was considered statistically significant.

## 3. Results

### 3.1. Expression of miR-21 in GC Specimens

**Figure 1:**
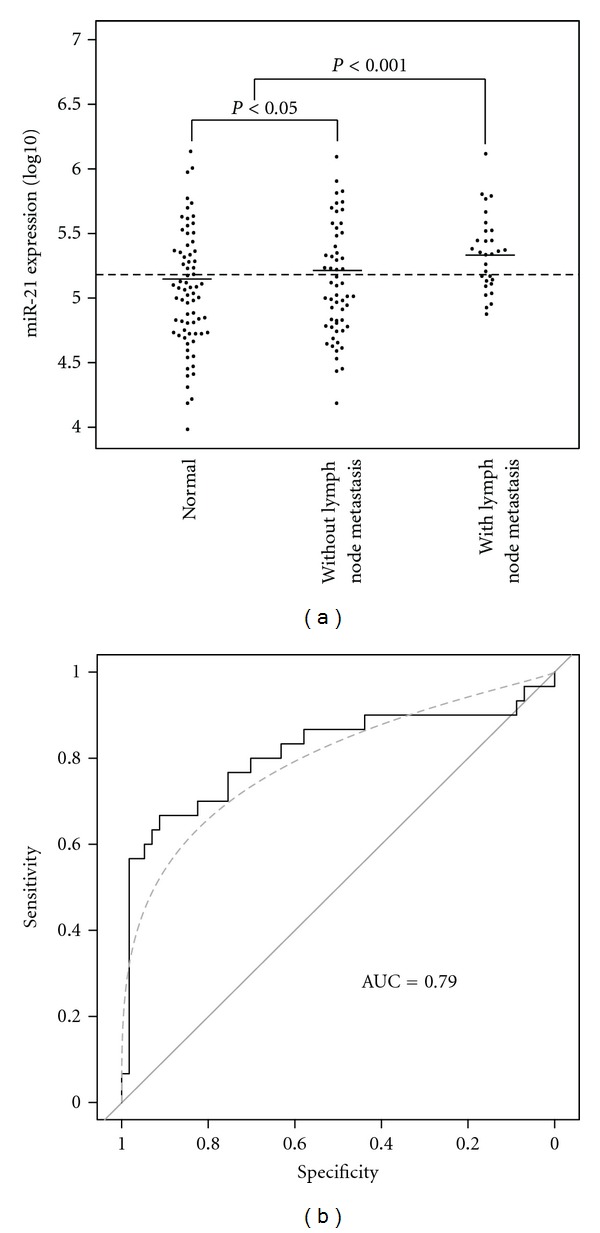
Comparison of levels of miR-21 in samples from gastric cancer (GC) patients without lymph node metastasis (*n* = 56), GC patients with lymph node metastasis (*n* = 30), and normal controls (*n* = 72). The lines denote the medians. Dot lines at the y-axis denote cut-off values. A significant difference between all histological groups was examined by ANOVA test. NS denotes no significant difference between groups. Receiver operating characteristics (ROCs) curves were used to discriminate patients with and without lymph node metastasis.

The expression level of miR-21 was analyzed by qRT-PCR. The expression level of internal control, Let-7a, showed no significant difference between the three groups (data not shown). We found significant difference between normal controls and patients without lymph node metastasis (*P* < 0.05). Furthermore, there is significant increase of miR-21 expression levels in patients with lymph node metastasis compared to normal controls and patients without lymph node metastasis (Figure 1(a)). A cut-off value of 5.12 was best distinguished in patients with lymph node metastasis and without lymph node metastasis, and the AUC value was 0.79 (Figure 1(b)). 

### 3.2. Correlation of miR-21 Expression with Clinicopathological Factors of GC Patients

The expression of miR-21 was found to be highly upregulated in 30 (34.9%) of 86 cases, whereas the remaining 56 cases (65.1%) were classified as having low up-regulated expression. To investigate the clinical significance of up-regulated miR-21, the relationship between miR-21 and clinicopathological factors was further assessed. As shown in Table 1, high up-regulated miR-21 expression appeared to be significantly associated with more histologic type (*P* < .001), T stage (*P* < .001), lymph node metastasis (*P* < .001) and pTNM stage (*P* < .05) in univariate analysis. Multivariate analysis found that pTNM stage (*P* = .001) and lymph node metastasis (*P* = .001) were statistically significant.

### 3.3. Correlation of miR-21 Expression with Prognosis of GC Patients

**Figure 2:**
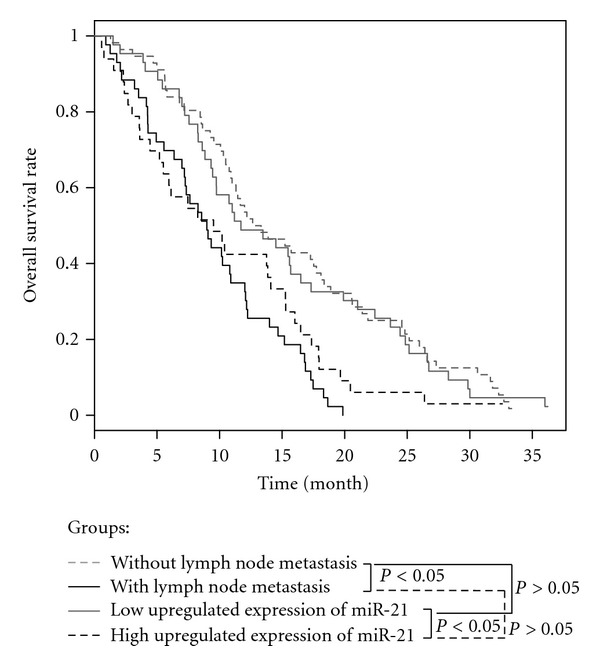
Kaplan-Meier survival curves of GC patients. The overall survival rate in patients with high miR-21 expression was significantly lower than that in those with low miR-21 expression (*P* < 0.05).

The overall survival rates in the patients with or without lymph node metastasis and with low or high up-regulated expression of miR-21 were statistically estimated. As shown in Figure 2, the median overall survival time in patients with high and low up-regulated miR-21 expression levels was nearly 11.2 and 13.8 months, respectively. The survival difference between these two groups was statistically significant (*P* < 0.05). We found that there were no significant differences between the patients with lymph node metastasis and high up-regulated expression of miR-21 and without lymph node metastasis and low up-regulated expression of miR-21 (*P* > 0.05). 

## 4. Discussion

To develop sensitive and specific minimally invasive molecular biomarkers for tailored management of cancers is a major challenge in clinical oncology. Dozens of studies have shown that miRNAs might be used as potential molecular biomarkers for human malignancies. Although numerous studies have been published for investigating the effect of the abnormal expression levels of miRNAs on the tumorigenesis, there is only a paucity of reports dealing with clinical impact of miRNAs in patients with lymph node metastasis [17–21]. Therefore, identification of miRNAs expression in patients with lymph node metastasis will be helpful to facilitate the clinical management of GC.

In this study, we have established the potentiality of miR-21 in the tumor tissue as a biomarker for lymph node metastasis. Using qRT-PCR, miR-21 was identified to be highly upregulated in GC patients with lymph node metastasis compared to patients with no lymph node metastasis (*P* < 0.05). ROC curve showed that the AUC value was nearly 0.8, which indicated that the expression level of miR-21 might be used to predict the lymph node metastasis in patients with GC. A previous study reported by Chan et al. also showed that miR-21 was overexpressed in most of the gastric cancer patients [22]. In addition, miR-21 and its precursor have been reported to be upregulated in many human malignancies, including pancreatic cancer, breast cancer, colorectal cancer, glioblastomas, and lung cancer [20, 23, 24]. 

The molecular mechanisms of regulating the expression of miR-21 in GC are rarely reported, especially the reports of evaluating the correlation between miR-21 expression level and clinical stage, lymph node metastasis, and prognosis of GC patients. The evidence was derived from the recently published reports. Suppression of miR-21 in MCF-7 cell, which overexpressed miR-21, could increase apoptosis and decrease cell proliferation, and knockdown of miR-21 in glioblastoma cells also showed that this miRNA has an anti-apoptotic function [25, 26]. Furthermore, two direct targets of miR-21, PDCD4 and maspin, which could decrease the metastasis of malignancies, have been found [27]. Taken other line of evidence together, a possible role of miR-21 as an oncogene has been hypothesized, including proliferation, cell cycle, metastasis, and chemosensitivity of tumor cells by targeting several tumor suppressor genes such as PTEN, MARCKS, PDCD4, and Cdc25A [28–31]. However, additional studies should be designed to investigate the molecular mechanisms of both the cause and effect of altered expression of miR-21 in GC.

Our present study next focused on the potential relationship between the expression level of up-regulated miR-21 and various GC clinicopathological factors, as well as the prognosis of the patients. It is worth noting that high up-regulated expression level of miR-21 was significantly correlated with histologic type, T stage, lymph node metastasis, pTNM stage, and poor overall survival of the patients with GC. High up-regulated expression of miR-21 in GCs with lymph node metastases indicates that its up-regulation was acquired in the course of tumor progression and, in particular, during the acquisition of metastatic potential. These results suggest that miR-21 could serve as a prognostic marker for prognosis of GC patients. However, the reports published by chan et al. did not show that higher expression of miR-21 affected the clinical prognosis of gastric cancer patients [22]. A potential important reason is that the patient cohort is too small to find the statistically significant difference, which only included 24 and 13 patients in group of <2-fold and ⩾2-fold elevated expression, respectively. Meanwhile, the design of our study was also different from the report by chan et al. In our study, we mainly examined the miR-21 expression in GC with lymph node metastasis, 56 and 30 GC patients without and with lymph node metastasis were included in the present study, respectively, and in the study reported by chan et al., only 2 and 35 GC patients without and with lymph node metastasis were included, respectively. Other researchers also reported the clinical significance of miR-21expression in human cancers. Yan et al. reported that miR-21 overexpression was correlated with specific breast cancer clinicopathological features, advanced tumor stage, lymph node metastasis, and poor survival of the patients [20]. Other reports showed that miR-21 expression in lung cancer was significantly correlated with advanced TNM stage, presence of lymph node metastasis, and overall survival [24]. This result indicates that miR-21 expression level could be used for the prediction of the clinical outcome. Of course, further prospective studies with a larger cohort are needed to confirm its prognostic significance in GC patients.

In conclusion, high up-regulated expression of miR-21 in GC was correlated with lymph node metastasis. Furthermore, the expression level of miR-21 might be also a potential prognostic factor for GC patients. Further studies are needed to verify the impact of miR-21 expression on gastric cancer, including the metastasis and prognosis.

## Figures and Tables

**Table 1:** Relationship between clinicopathological factors and miR-21 expression in patients with GC.

Parameters	miR-21 expression		*P* value^∗^
	Low (*n* = 56)	High (*n* = 30)
Age (means ± SD, years)	56.03 ± 12.86	56.26 ± 11.14	0.436
Gender			0.128
Male	35 (62.3%)	20 (66.1%)	
Female	21 (37.7%)	10 (33.9%)	
Tumor location			0.526
Proximal	3 (5%)	1 (3%)	
Body	24 (42.6%)	12 (41%)	
Distal	28 (50.4%)	17 (56%)	
Histologic type			<0.001
Differentiated	19 (34.3%)	7 (22.5%)	
Undifferentiated	37 (65.7%)	23 (77.5%)	
T stage			<0.001
T1	37 (66.7%)	15 (49.3%)	
T2	12 (21.4%)	7 (23.6%)	
T3	7 (11.9%)	5 (18.1%)	
T4	0 (0%)	3 (9%)	
Lymph node metastasis			<0.001
Present	17 (30.5%)	26 (85.3%)	
Absent	39 (69.5%)	4 (14.7%)	
Liver metastasis			0.266
Present	27 (47.8%)	15 (50.3%)	
Absent	29 (52.2%)	15 (49.7%)	
Peritoneal metastasis			0.137
Present	32 (57.8%)	18 (61.2%)	
Absent	24 (42.2%)	12 (38.8%)	
pTNM stage			<0.05
I	29 (52.6%)	12 (41.1%)	
II	16 (27.8%)	7 (21.8%)	
III	6 (11.3%)	6 (19.3%)	
IV	5 (8.3%)	5 (17.8%)	

*P value was tested in univariate analysis.
